# Exploring the association between herbal medicine usage and drug-induced liver injury: insights from a nationwide population-based cohort study using SCCS in South Korea

**DOI:** 10.3389/fphar.2025.1498124

**Published:** 2025-01-29

**Authors:** Taehyun Yang, Juhee Ahn, Sungho Won, Sanghun Lee

**Affiliations:** ^1^ Department of Public Health Sciences, Graduate School of Public Health, Seoul National University, Seoul, Republic of Korea; ^2^ Institute of Health and Environment, Seoul National University, Seoul, Republic of Korea; ^3^ RexSoft Inc, Seoul, Republic of Korea; ^4^ Department of Bioconvergence and Engineering, Dankook University, Gyeonggi-do, Republic of Korea

**Keywords:** drug-induced liver injury, herbal medicines, traditional Korean medicines, herbal and dietary supplements, pharmacovigilance, hepatotoxicity, self-controlled case series

## Abstract

**Introduction:**

Drug-induced liver injury (DILI) is a significant health concern caused by exposure to pharmaceuticals, over-the-counter medications, herbal remedies, and dietary supplements. The contribution of prescribed herbal medicines to DILI risk remains unclear. This study aimed to evaluate the hepatotoxicity risk associated with traditional Korean medicines (TKMs) using nationwide health insurance claims data.

**Methods:**

A tailored cohort of patients diagnosed with DILI (ICD-10 code: K71) between January 2011 and December 2019 was obtained from the Health Insurance Review and Assessment Service. After applying inclusion and exclusion criteria, 672,411 patients were identified. Using a self-controlled case study (SCCS) design, exposures were defined as hospital/clinic visits or medication prescriptions within a 90-day window. Analyses were conducted across three groups: outpatients, inpatients, and patients with liver disease. Relative incidences of DILI were calculated for different exposure scenarios.

**Results:**

Outpatients showed the highest relative incidences of DILI 3–15 days after visiting Western hospitals/clinics or being prescribed commercial drugs, with risk estimates of 1.55 (95% confidence interval [CI]: 1.55–1.56) and 2.44 (95% CI: 2.43–2.44), respectively. These risks gradually declined to baseline levels (1.0). All other groups exhibited similar patterns. In contrast, DILI risks associated with TKM hospital/clinic visits and herbal medicine prescriptions were minimal, with relative risks of 1.01 (95% CI: 1.00–1.01) and 0.99 (95% CI: 0.99–0.99), respectively. However, a mildly elevated risk was observed in patients with liver disease.

**Conclusion:**

This nationwide cohort study demonstrates that herbal medicines prescribed by TKM practitioners have minimal impact on DILI risk. Patients with pre-existing liver disease exhibit increased susceptibility to DILI. Differentiating between unregulated herbal products and those prescribed in medical institutions is essential for accurate assessment of hepatotoxicity risk.

## Introduction

Drug-induced liver injury (DILI) is a condition in which the liver is damaged due to exposure to prescription pharmaceuticals, over-the-counter medicines, and herbal and dietary supplements (HDS) (Real et al., 2019). It is characterized by a range of liver abnormalities, varying from mild elevations in liver enzyme levels (transaminases) to severe liver damage or failure, which can be life-threatening (Real et al., 2019; Nunes et al., 2022). The incidence of DILI is increasing worldwide because of the prevalence and widespread use of healthy functional foods and self-medication, available from a variety of sources.

Recent studies have highlighted the increasing DILI risk posed by HDS, alongside traditional risk factors such as antibiotics and anti-inflammatory drugs (Real et al., 2019; Nunes et al., 2022). In South Korea, reports have presented conflicting findings regarding the primary causative agents of DILI, particularly concerning herbal medicines prescribed by traditional Korean medicine (TKM) doctors (Kim et al., 2003; Yoo et al., 2007; Suk et al., 2012). Initial reports in 2003 suggested a significant association between herbal medicines and DILI, accounting for 57.9% of cases (Kim et al., 2003); subsequent studies in 2007 and 2012 also found they accounted for approximately 30% of cases (Yoo et al., 2007; Suk et al., 2012). However, reports from multi-pharmacovigilance centers in South Korea have revealed that antibiotics, anti-epileptics, anti-inflammatory drugs, and statins are the major agents associated with DILI (>80%), whereas herbal medicines accounted for only 0.5% of cases (Shin et al., 2009; Kwon et al., 2012). Retrospective studies among patients taking herbal medicines prescribed by TKM doctors also indicated a low DILI prevalence (∼0.5%), with subclinical or mild symptoms (Lee A. R. et al., 2012; Lee et al., 2015). Additionally, a nationwide prospective study conducted between April 2013 and January 2016 estimated the incidence of DILI from herbal medicines to be 0.6% (Cho et al., 2017).

Taken together, the safety profiles of herbal medicines in South Korea exhibit discrepancies (Lee et al., 2019). It is imperative to conduct a nationwide population-based study encompassing all DILI cases to address the limitations inherent in relying solely on studies conducted in Western or TKM institutions. Given the comprehensive healthcare coverage provided by South Korea’s universal healthcare system, robust data from nationwide health insurance claims are readily accessible through the Health Insurance and Review Assessment (HIRA) process.

Here, we adopted the self-controlled case series (SCCS) due to several compelling reasons. First and foremost, SCCS effectively controls for all confounders that do not vary over time within an individual, such as genetic factors, lifestyle choices, and long-term health conditions (Petersen et al., 2016). This is crucial in our study where such confounders could significantly impact the results. By using SCCS, we minimize bias arising from these time-invariant confounders, ensuring more accurate and reliable findings. Moreover, SCCS is highly efficient as it only includes individuals who have experienced the event of interest—in this case, DILI (Lee C. H. et al., 2012; Brauer et al., 2016). This allows for a focused and efficient analysis, which is particularly valuable given the potential rarity of DILI events. Another key reason for the necessity of SCCS in our study is its ability to minimize selection bias (Mostofsky et al., 2018). In traditional cohort or case-control studies, there is a risk of selection bias due to differences between exposed and non-exposed groups. SCCS mitigates this risk by using each individual as their own control, thereby enhancing the validity of our findings. By SCCS, we investigated the relative incidence of DILI associated with exposure to TKM hospitals/clinics and herbal medicines prescribed by TKM doctors 15, 30, 45, 60, 75, and 90 days before the onset of DILI symptoms.

## Materials and methods

### Data source

The nationwide health claims database provided by the HIRA in South Korea between January 2011 and December 2019 was used, which was chosen to ensure the availability and completeness of the most recent, high-quality data. The data comprised three parts: general information on individuals, all electronically submitted diagnoses (based on the International Classification of Diseases 10th Revision; ICD-10), and medication prescriptions (including herbal medicines). The study protocol was reviewed by the Institutional Review Board of Dankook University (Approval Number: DKU 2020-09-001). Access to and use of the HIRA database were authorized by HIRA through a remote-controlled desktop (Approval Number: HIRA M20200924766), ensuring compliance with ethical guidelines and data protection regulations throughout the study period.

### Study population

Data on 707,365 individuals who developed DILI from January 2011 to December 2019 was obtained. DILI was defined as toxic liver disease (ICD-10 code: K71). Recurrent episodes were excluded from the analysis to ensure adherence to the SCCS assumptions, and 34,954 cases from 2011 were excluded because DILI episodes before 2011 were not considered, resulting in a final dataset of 672,411 individuals.

**FIGURE 1:**
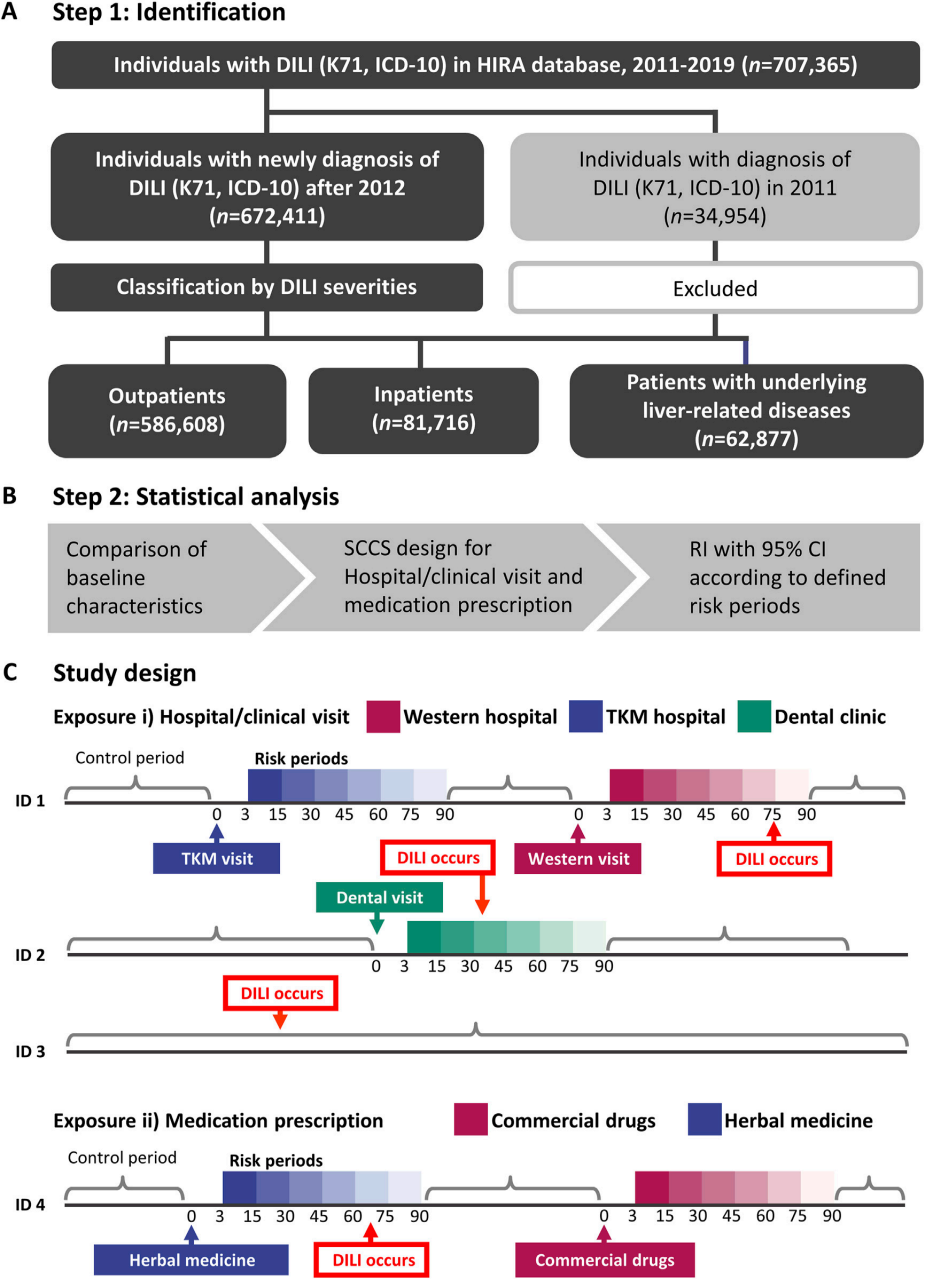
The flowchart for the study population inclusion/exclusion and the final three groups **(A)**, analysis flow for self-controlled case series **(B)**, and the detailed study design **(C)**. Risk of hospital visit or prescription and the control period of patients were defined. DILI, drug-induced liver injury; HIRA, Health Insurance and Review Assessment; ICD-10, International Statistical Classification of Diseases, 10th Revision; RI, relative incidence; TKM, traditional Korean medicine; SCCS, self-controlled case series.

To categorize DILI severity, the study population was divided into three groups: outpatients (individuals who received a DILI diagnosis and were not admitted to hospital), inpatients (individuals admitted to hospital at the time of DILI diagnosis), and patients with liver disease (a high-risk DILI group comprising individuals with pre-existing liver conditions such as hepatitis, malignant neoplasm of liver and intrahepatic bile ducts, alcoholic liver disease, hepatic failure, fibrosis and cirrhosis of liver, and other inflammatory liver diseases; detailed in Supplementary Table S1). The investigation workflow is illustrated in Figure 1A.

### Study design

A SCCS design was used to mitigate the impact of time-invariant confounding variables (Figures 1B, C). Age and exposure were treated as time-variant variables. Exposures were defined as i) hospital/clinic visits or ii) prescriptions, respectively. Specifically, hospital/clinical visit refers to any visit, whether or not medications were prescribed. Although data on most medications prescribed in Western medical institutions were available in the nationwide health claims database, 56 herbal extracts prescribed in TKM institutions were collected. Therefore, a visit to either a Western or TKM institutions could be conservatively considered as exposures to commercial drugs or herbal medicines.

For both exposure types, risk periods were defined within 15, 30, 45, 60, 75, and 90 days following each exposure to observe the temporal pattern of DILI incidence. This enabled an evaluation of the consistency and time of DILI relative to initial exposure. The primary outcome was the first occurrence of DILI, coded as K71. To reduce the potential for the reverse causation between exposure and outcome, K71 events occurring within 0–2 days after exposure were excluded. Cases where patients had concurrent exposures to both herbal and conventional medicines within the same risk period were excluded from the analysis to avoid potential confounding effects.

### Statistical analysis

Demographic and clinical characteristics are reported as mean ± standard deviation for continuous variables and as frequencies and percentages for categorical variables. The key assumptions of the SCCS model included the independence of recurrent outcome events, where the occurrence of one event does not influence the probability of subsequent events or the independence of outcome events from subsequent exposure (Petersen et al., 2016). The first occurrence of DILI was focused on, for which the estimated relative incidence (RIs) may be conservative. RIs with 95% CIs in comparison with the control period were separately estimated according to defined risk periods (Figure 1C). Statistical analyses were performed using R (version 4.3.1) with the SCCS package of the R Foundation for Statistical Computing and SAS Enterprise Guide (version 7.13; SAS Institute, Cary, NC, United States) (Farrington et al., 2018).

## Results

### Study population and characteristics

A total of 586,608 outpatients (Group A), 85,803 inpatients (Group B), and 62,877 patients with liver disease (Group C) were included; demographic and clinical characteristics are available in Table 1. Across all groups, the average age was approximately 50 years, with a higher proportion of males than females; Group C had the highest number of male patients (61.38%). The majority of Group A received their diagnosis at primary care institutions (58.96%), whereas most inpatients were diagnosed at secondary or tertiary care hospitals (73.32% and 23.24%, respectively). Mortality rates were highest in Group B, indicating more severe DILI. Group C had a higher prevalence of comorbidities, with dyslipidemia being the most common. The predominant subtype of DILI in Group C was toxic liver disease with cholestasis (45.27%), whereas unspecified DILI was more prevalent in Groups A and B (>55%).

**TABLE 1:** Patient demographics and clinical features at diagnosis of drug-induced liver injury (DILI) for outpatients, inpatients, and patients with liver diseases.

	Outpatients	Inpatients	Patients with liver diseases
Patients (number)	586,608	85,803	62,877
Males; [n (%)]	299,844 (51.14)	47,686 (55.58)	38,592 (61.38)
Age (mean ± SD)	50.15 ± 16.87	51.03 ± 19.40	50.50 ± 15.73
Subtype of toxic liver disease			
Unspecified (K71.9)	326,217 (55.61)	47,938 (55.87)	3,630 (5.77)
Acute hepatitis (K71.2)	99,051 (16.88)	19,617 (22.86)	10,958 (17.43)
Hepatitis, not elsewhere classified (K71.5)	2,130 (0.36)	497 (0.58)	1,013 (1.61)
Cholestasis (K71.0)	26,137 (4.45)	2,099 (2.44)	28,465 (45.27)
Medical Institution of Diagnosis			
Primary care institution	345,868 (58.96)	1,700 (1.98)	21,485 (34.17)
Secondary care hospital	175,630 (29.94)	62,909 (73.32)	28,456 (45.26)
Tertiary care hospital	63,407 (10.81)	19,941 (23.24)	12,718 (20.23)
Comorbidity			
Hypertension	36,513 (6.22)	885 (1.03)	4,861 (7.73)
Type 2 Diabetes Mellitus	33,338 (5.68)	2,090 (2.44)	4,670 (7.43)
Dyslipidemia	73,347 (12.50)	649 (0.76)	11,477 (18.25)
Length of hospital stay (days)	0.99 ± 0.04	8.55 ± 8.16	2.06 ± 3.99
Mortality (%)	59 (0.01)	1,140 (1.33)	276 (0.44)

Data are reported as mean ± standard deviation or number (percentage).

### DILI incidence risk assessment after different medical institution visits

**FIGURE 2:**
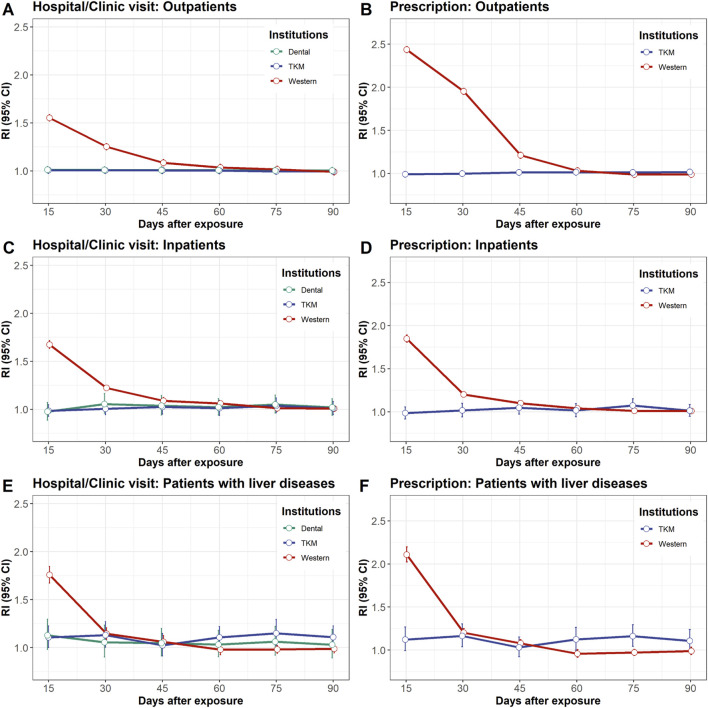
Relative incidence of drug-induced liver injury after exposure to hospital/clinical visits left panel: **(A, C, E)** or prescriptions right panel: **(B, D, F)**. The study population was divided into three groups: outpatients upper panel: **(A, B)**, inpatients middle panel: **(C, D)**, and patients with liver disease lower panel: **(E, F)**. Exposure to hospital/clinical visit refers to any visit, with or without prescriptions for commercial drugs or herbal medicines. CI, confidence interval; RI, relative incidence.

The risks of DILI associated with each medical institution are shown in Figures 2A–C. The risks within 3–15 days following visits to Western hospitals/clinics were consistently higher than those for visits to other medical institutions across Group A, B, and C (RI = 1.55 [95% CI: 1.55–1.56]; RI = 1.67 [95% CI: 1.65–1.70]; RI = 1.76 [95% CI: 1.71–1.81], respectively). The elevated RIs converged to 1.0 after 3–15 days, indicating a decrease in the risk of DILI over time.

In contrast, no elevated risk of DILI was observed in Group A and Group B within 3–15 days following visits to TKM hospitals/clinics (RI = 1.01 [95% CI: 1.00–1.01] and RI = 0.98 [95% CI: 0.93–1.05], respectively) (Figures 2A, B). This trend persisted across the other risk periods for up to 90 days. Similarly, the RIs of DILI for all risk periods associated with visits to dental hospitals/clinics were close to 1.0, indicating minimal risk. In Group C, the risk of DILI was mildly elevated 61–75 days after visits to TKM hospitals/clinics (RI = 1.15 [95% CI: 1.05–1.25]) (Figure 2C).

### DILI incidence risk assessment after prescription (commercial drug or herbal medicine)

The risk of DILI from prescription is shown in Figures 2D–F. The risks within 3–15 days following prescription of commercial drugs were consistently higher compared to herbal medicine prescribed by TKM doctors across Groups A, B, and C (RI = 2.44 [95% CI: 2.43–2.44]; RI = 1.85 [95% CI: 1.83–1.87]; RI = 2.11 [95% CI: 2.07–2.15], respectively). The elevated RIs gradually converged to 1.0 after 16 days, indicating a decrease in the risk of DILI over time.

Conversely, there was no elevated risk of DILI within 3-15 days following herbal medicine prescription in Group A or B (RI = 0.99 [95% CI: 0.99–1.00] and RI = 0.98 [95% CI: 0.92–1.06], respectively) (Figures 2D, E). In Group C, the risk of DILI was mildly elevated at 16–30 and 61–75 days following herbal medicine prescription (RI = 1.16 [95% CI: 1.05–1.28] and RI = 1.16 [95% CI: 1.05–1.27], respectively) (Figure 2F).

## Discussion

This study established that visits to TKM institutions or prescriptions of herbal medicines, both for outpatients and inpatients populations, were associated with a negligible risk of DILI in a large-scale, population-based cohort. In contrast, a prominently elevated RI of DILI was observed following visits to Western institutions or prescriptions of commercial drugs. Furthermore, patients with pre-existing liver disease experienced a modest increase in DILI risk within 90 days of exposure to either hospital/clinic visit or prescriptions.

The risk factors for DILI remain poorly understood because most idiosyncratic cases are unpredictable and occur within the therapeutic doses of prescribed medication, indicating the absence of a dose-response relationship (Hoofnagle and Bjornsson, 2019). The pathophysiology of DILI is profoundly influenced by multiple variables including individual characteristics and environmental factors (Hussaini and Farrington, 2007; Chalasani and Bjornsson, 2010). Traditional epidemiological study designs, such as cohort and case-control studies, often struggle to adequately account for the myriad confounding variables inherent in DILI cases, particularly because identifying suitable control groups is challenging (Lee C. H. et al., 2012; Petersen et al., 2016). Determining the exposure timing to a candidate substance in DILI cases poses a pivotal challenge (Mostofsky et al., 2018). The SCCS design is particularly well suited for analyzing the impact of herbal medicines on DILI, addressing these complexities (Petersen et al., 2016; Nunes et al., 2022) However, the SCCS method also has some weaknesses (Mostofsky et al., 2018; Takeuchi et al., 2018) It requires accurate timing of both the exposure (such as herbal medicine usage) and the event (DILI), as misclassification can lead to biased results. Additionally, while SCCS controls for time-invariant confounders, it does not automatically control for time-varying confounders, which can introduce bias if not properly accounted for. There can also be issues with reverse causality, where the outcome might influence the exposure timing, which needs to be carefully considered and addressed in the study design. Lastly, if there are underlying temporal trends in the occurrence of the event or exposure, these need to be accounted for to avoid biased estimates.

In this study, both visitation and prescription of drugs in Western institutions resulted in a significantly higher risk of DILI across all groups. The sequentially decreasing risk from 3 to 15 days following exposure suggests that both these factors influence DILI development. In contrast, inpatients and outpatients visiting and/or prescribed medicine at TKM institutions showed a minimal risk of DILI, though increased risk was observed within 75 days of exposure in patients with liver diseases. This indicates that obtaining patient histories is essential before prescription by TKM physicians.

Previous studies in East Asian countries have also had controversial results regarding the effects of herbal medicines on DILI. In Taiwan, a population-based cohort study highlighted acetaminophen (35.0%) and anti-tuberculous drugs (34.7%) are major causes of DILI, whereas herbal medicines were not (Sobhonslidsuk et al., 2016). Another prospective study in Taiwan from 2011 to 2019 suggested 78.0% of DILI cases were caused by conventional drugs, with 22.0% caused by HDS, but included diverse nutritional supplements without prescriptions (Huang et al., 2021). In mainland China, a retrospective study identified Traditional Chinese Medicine (TCM) or HDS as the leading cause of DILI, accounting for 26.81% of cases (Shen et al., 2019). However, a significant methodological concern has been raised regarding the study by Shen et al. (Shen et al., 2019), particularly the decision to classify TCM and HDS as a single category (Cong et al., 2019; Yang et al., 2019). TCM, like TKM, is a highly regulated practice, prescribed by licensed practitioners, which is in stark contrast to HDS, a category encompassing a wide array of products with varying degrees of regulation and quality. By combining these distinct entities into one category, the study oversimplifies the data, potentially exaggerating the risk associated with TCM while obscuring the specific dangers posed by less regulated HDS products. This flawed classification results in an unequal comparison with conventional drugs, possibly leading to misleading conclusions about the main contributors to DILI. For a more accurate analysis, TCM and HDS should be categorized separately, with further subcategories to account for their diversity and regulatory differences. Overall, the clear hepatotoxicity risks associated with unregulated herbal medicines emphasizes the need for these products to prescribed within medical institutions, as advocated by our study.

This study had several limitations. First, the K71 code used to identify cases of DILI encompasses both idiosyncratic and intrinsic forms of liver injury, and we were unable to perform further distinctions or stratified analyses due to the absence of relevant information, such as laboratory data, in the HIRA database (Kim et al., 2017). The reliance on ICD-10 codes, which lack detailed clinical information, may have led to the potential misclassification of DILI cases compared to diagnoses established through clinical evaluation. As a result, we were unable to apply the Roussel Uclaf Causality Assessment Method (RUCAM) causality grading (Roussel Uclaf Causality Assessment Method (RUCAM) in Drug Induced Liver Injury (2012)) to confirm the association between exposure and DILI events. Second, the HIRA database does not include details on specific medications indicating the precise causes of liver injury. Information on herbal medicines in the HIRA database was restricted to insurance-covered extracts, excluding decoctions and non-insured herbal formulations, which are commonly used in practice. The limited scope of data on non-insured herbal medicines introduces potential bias and restricts the generalizability of our findings. Consequently, we could not clearly distinguish herb-induced liver injury from other forms of DILI within the K71-coded events. Third, the observed IRs within 3–15 days of exposure in Western institutions may have been overestimated due to the case-only design, which focused on the first episode of DILI. However, the elevated risk observed during the subsequent 16–30 days, followed by a decline, supports the temporal association. As a reference point, visits to dental clinics showed no associated risk for any group throughout the study period, reinforcing the reliability of the findings. Lastly, pre-existing liver disease has been reported to result in more severe outcomes upon the occurrence of DILI rather than influencing its incidence (Chalasani and Bjornsson, 2010). However, the risk associated with specific herbal components in patients with liver disease could not be evaluated.

Future research on herbal medicine safety should adopt a multifaceted approach by integrating electronic medical records with health insurance claims data to identify specific herbs or formulations that may pose higher risks for DILI. This integration would provide a more comprehensive understanding by combining clinical observations with prescription data. Additionally, network pharmacology approaches should be employed to predict hepatotoxicity risks of individual herbs or compounds (Hong et al., 2017), uncovering potential interactions and aiding in proactive risk management strategies. Regulatory and legislative support is essential to establish robust pharmacovigilance systems for herbal prescriptions, including real-time monitoring, reporting, and risk mitigation mechanisms. Furthermore, future studies should focus on the safety profiles of specific herbs, particularly their effects on vulnerable populations, such as individuals with pre-existing liver conditions or those using concurrent medications, to address existing knowledge gaps and enhance the safe use of herbal medicines in clinical practice.

In conclusion, this study highlights a significant association between commercial drugs and DILI incidence, while confirming that herbal medicines prescribed by TKM doctors have minimal impact on DILI risk. These findings contribute to our understanding of the DILI risks associated with herbal medicine, particularly by emphasizing the heightened vulnerability of patients with liver diseases. Moreover, they underscore the necessity for further research into the risk factors underlying DILI development. Additionally, there is an urgent demand to assess the hepatotoxicity risk posed by unregulated herbal products in comparison to herbal medicines prescribed within medical institutions.

## Data Availability

Publicly available datasets were analyzed in this study. This data can be found here: HIRA database. Access to and use of the HIRA database were authorized by HIRA through a remote-controlled desktop (Approval Number: HIRA M20200924766).
