# Supplementary material for: Exploring the association between herbal medicine usage and drug-induced liver injury: insights from a nationwide population-based cohort study using SCCS in South Korea
Source: Front Pharmacol. 2025 Jan 29;16:1498124. doi: 10.3389/fphar.2025.1498124 (PMC11813889; doi:10.3389/fphar.2025.1498124)
Supplement: Supplementary file 1 [file Table1.docx]

**Supplementary Material**

| **Table S1.** Definition of liver diseases according to ICD-10 codes. | **p. 01** |
| --- | --- |

Table S1 Definition of liver diseases according to ICD-10 codes.

| **ICD-10 code** | **Definition** |
| --- | --- |
| B17–B19, B25.1, B58.1, K73 | Hepatitis |
| C22 | Malignant neoplasms of liver and intrahepatic bile ducts |
| K70 | Alcoholic liver disease |
| K72 | Hepatic failure |
| K74 | Fibrosis and cirrhosis of liver |
| K75 | Other inflammatory liver disease |

ICD-10, International classification of diseases, tenth revision.
